# LocTree3 prediction of localization

**DOI:** 10.1093/nar/gku396

**Published:** 2014-05-21

**Authors:** Tatyana Goldberg, Maximilian Hecht, Tobias Hamp, Timothy Karl, Guy Yachdav, Nadeem Ahmed, Uwe Altermann, Philipp Angerer, Sonja Ansorge, Kinga Balasz, Michael Bernhofer, Alexander Betz, Laura Cizmadija, Kieu Trinh Do, Julia Gerke, Robert Greil, Vadim Joerdens, Maximilian Hastreiter, Katharina Hembach, Max Herzog, Maria Kalemanov, Michael Kluge, Alice Meier, Hassan Nasir, Ulrich Neumaier, Verena Prade, Jonas Reeb, Aleksandr Sorokoumov, Ilira Troshani, Susann Vorberg, Sonja Waldraff, Jonas Zierer, Henrik Nielsen, Burkhard Rost

**Affiliations:** 1 Department of Informatics, Bioinformatics-I12, TUM, 85748 Garching, Germany; 2 TUM Graduate School, Center of Doctoral Studies in Informatics and its Applications (CeDoSIA), 85748 Garching, Germany; 3 Biosof LLC, New York, NY 10001, USA; 4 Center for Biological Sequence Analysis, Department of Systems Biology, DTU, 2800 Lyngby, Denmark; 5 Institute for Advanced Study (TUM-IAS), 85748 Garching, Germany; 6 New York Consortium on Membrane Protein Structure (NYCOMPS) & Department of Biochemistry and Molecular Biophysics, Columbia University, New York, NY 10032, USA; 7 Institute for Food and Plant Sciences WZW – Weihenstephan, 85350 Freising, Germany

## Abstract

The prediction of protein sub-cellular localization is an important step toward elucidating protein function. For each query protein sequence, LocTree2 applies machine learning (profile kernel SVM) to predict the native sub-cellular localization in 18 classes for eukaryotes, in six for bacteria and in three for archaea. The method outputs a score that reflects the reliability of each prediction. LocTree2 has performed *on par with* or better than any other state-of-the-art method. Here, we report the availability of LocTree3 as a public web server. The server includes the machine learning-based LocTree2 and improves over it through the addition of homology-based inference. Assessed on sequence-unique data, LocTree3 reached an 18-state accuracy *Q*18 = 80 ± 3% for eukaryotes and a six-state accuracy *Q*6 = 89 ± 4% for bacteria. The server accepts submissions ranging from single protein sequences to entire proteomes. Response time of the unloaded server is about 90 s for a 300-residue eukaryotic protein and a few hours for an entire eukaryotic proteome not considering the generation of the alignments. For over 1000 entirely sequenced organisms, the predictions are directly available as downloads. The web server is available at http://www.rostlab.org/services/loctree3.

## INTRODUCTION

Many experimental methods annotate protein localization, enriching resources such as SWISS-PROT (1). However, even for the well-studied yeast, the experimental data are not nearly complete (2,3). Bridging the sequence-annotation gap (4) for localization, therefore, calls for cheaper and faster *in silico* approaches (5,6). Many machine learning methods predict the native localization of a protein from its amino acid sequence; among the best known are CELLO (7), WoLF PSORT (8), YLoc (9) and PSORTb (10). A recent study suggested homology-based inference to outperform machine learning (11). Homology-based inference proceeds as follows: build a data set with all proteins of known localization, run a simple pairwise BLAST (12) against this set, and predict the localization of the first hit.

LocTree2 predicts a single localization for all proteins in all domains of life through machine learning (13). The method implements a hierarchical system of Support Vector Machines (SVMs) to imitate the cascading mechanism of cellular sorting (14). An independent, recent benchmark proved LocTree2 to be an excellent successor and/or complement to other top-of-the-line prediction methods (15) in situations in which no experimental information is available for the query protein or its homologs.

Here, we introduce LocTree3. It provides the web server front end for LocTree2, and improves over LocTree2 by including information about homologs if available. Thereby, LocTree3 combines ‘the best of both worlds’, employing homology when possible and machine learning otherwise. The major steps of improvement are as follows: (i) inclusion of annotation transfer from close homologs with experimentally annotated localization through PSI-BLAST (12); (ii) runtime reduction of LocTree2 by using a new fast implementation of the SVM profile kernel (16,17); (iii) Gene Ontology (18) annotations for prediction results; (iv) caching of the results for faster processing of the repeated searches (19,20).

## MATERIALS AND METHODS

### Data

The number of proteins with experimental annotation for a single localization in SWISS-PROT release 2011_04 was 34 583 for eukaryotes (18 localization classes, visualized in Figure 2), 4765 for bacteria (six classes: cytosol, plasma membrane, periplasmic space, outer membrane, fimbrium and extra-cellular) and 237 for archaea (three classes: cytosol, plasma membrane and extra-cellular). LocTree2 was developed on sequence-unique subsets with 1682 eukaryotic, 479 bacterial and 79 archaeal proteins (Supplementary Table S1, Supporting Online Material). Sequence-redundancy was reduced at HVAL ≤ 0 (21,22) through UniqueProt (23). This is commonly done because the bias in data sets from sequence similarity often overestimate performance (24). However, in order to assess the power of homology-based inference, we had to accept some redundancy because homology-based inference performed below the level of random across sequence-unique proteins (Supplementary Table S2). We accomplished this by running the sequence-unique 1682 eukaryotic proteins against all experimentally annotated proteins, i.e. against the same release of SWISS-PROT putting the redundancy back in to enable PSI-BLAST lookups. For 995 of the 1682, PSI-BLAST found a non-trivial (removal of query protein) at E-value ≤ 10^−3^ (25,26); for 687 it did not.

**Figure 1.**
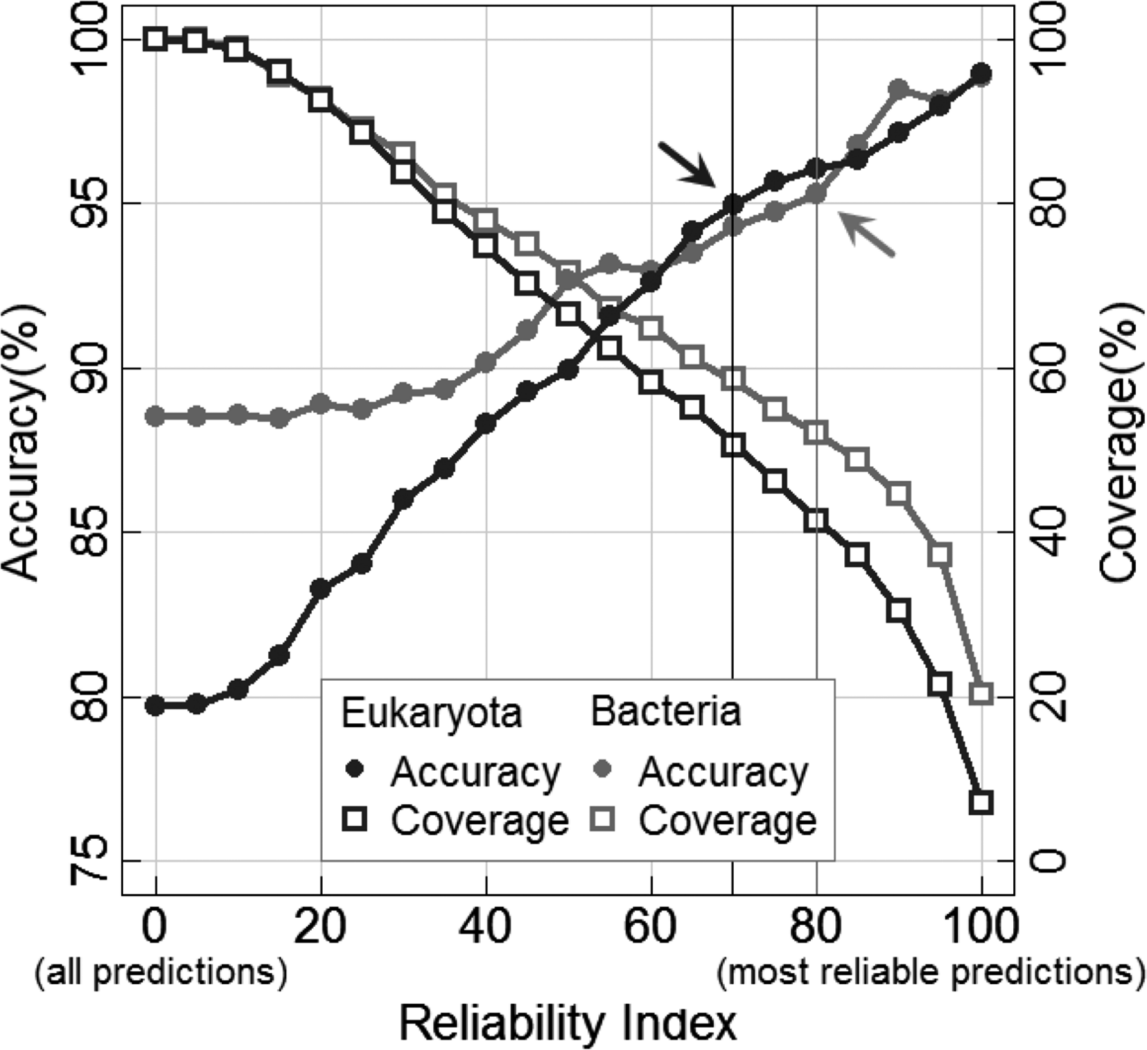
Reliable predictions more accurate. The reliability index (RI) of LocTree3 relates the strength of a prediction to the performance. The curves show the percentage accuracy/coverage (‘Materials and Methods’ section) for LocTree3 predictions above a given RI. Increasing the RI implies that we look at some subset of all predictions; the subset is given by the curves with squares. For instance, half of all eukaryotic proteins are predicted at RI > 70 (black cross-line). For this top 50%, performance rises from the average *Q*18 = 80% to *Q*18 = 95% (black line with circles, black arrow). Similar values are reached for RI > 80 for bacteria (gray cross-line; note that in this case *Q*6 = 95% is a six-state accuracy as opposed to the 18-state value for eukaryotes).

**Figure 2.**
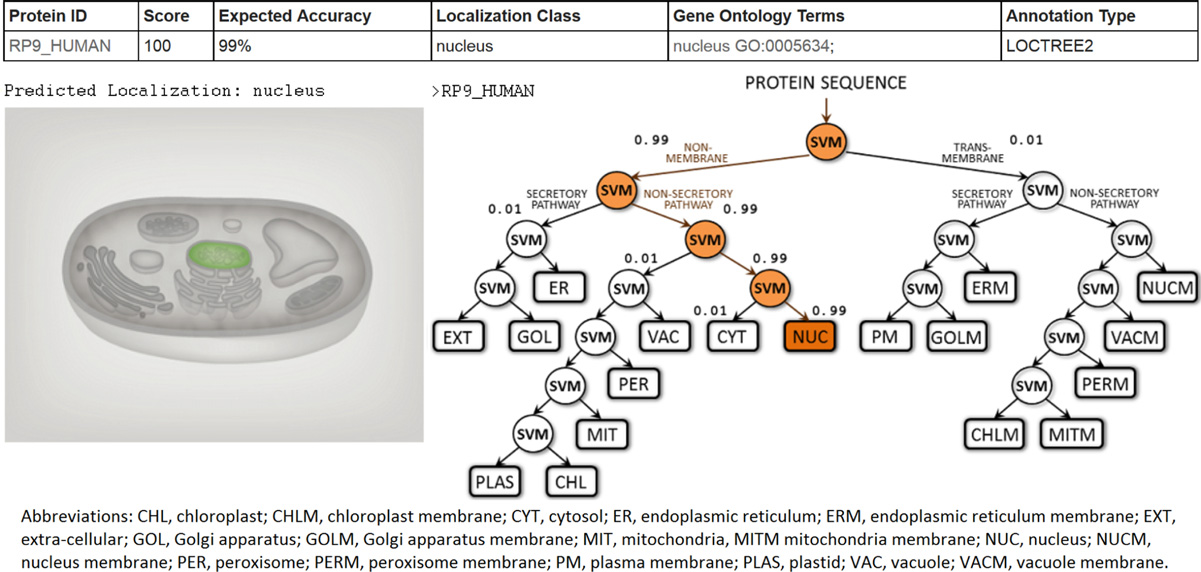
Example output for protein RP9_HUMAN. For every input protein sequence the LocTree3 prediction result contains: (i) protein identifier, (ii) reliability index, (iii) expected accuracy of the prediction, (iv) localization class, (v) GO term(s) and identifier(s) and (vi) source of the prediction. The predicted localization is highlighted in the schematic representation of the cell (here: nucleus). For LocTree2 predictions (shown here), we provide a visualization of the decision tree and the decision path leading to the final prediction. The reliability index is formed through the product of values along the decision path. For PSI-BLAST predictions, we provide a sequence alignment of the query protein to its best hit instead of the tree.

For further testing, we added three new data sets. We collected all proteins for which experimental annotations had been added between releases 2011_04 and 2013_11. We redundancy reduced those at HVAL ≤ 0. This gave the sets New2013_hval0 (273 for eukaryotes, 57 for bacteria). Additional redundancy reduction to LocTree3 development data provided too small sets (32 eukaryotic and two bacterial proteins) for reliable performance estimates. Next, we simulated the question ‘how well the method will perform on the next 1000 new proteins?’ by simply monitoring all proteins added since we began collecting the data for this manuscript, i.e. the proteins added since 2013_11 (New2014 with 198 eukaryotic proteins and too few in bacteria to proceed). Finally, we investigated a third set with all human proteins (Supplementary Table S3). We deliberately kept the ‘redundancy’ in this set that exists on the level of an organism. Note that throughout we have considered only proteins with single experimental annotations. Our preliminary analysis of proteins with multiple annotations suggested these to constitute a small set of proteins with many problematic annotations (Supplementary Section S1).

### Methods

1. **Homology-based inference:** We transferred localization annotations by homology through PSI-BLAST (12). For all proteins with experimentally known localization, we generated PSI-BLAST profiles using an 80% non-redundant database combining UniProt (1) and PDB (27) with two iterations and *E*-value ≤ 10^−3^. These profiles were then aligned against all proteins with experimental annotation of a single localization in SWISS-PROT release 2011_04. PSI-BLAST hits to the input protein were excluded.
2. **LocTree2** (13) utilizes a hierarchical system of SVMs. At all levels of the tree are binary decisions, which are made by searching through proteins of annotated localization with short stretches of *k*-consecutive residues (*k* = 3 for archaea, 5 for bacteria and 6 for eukaryota). The most informative *k*-mer hit decides on ‘left or right’ for each fork in the tree until reaching a leaf, i.e. the final predicted localization class.
3. **LocTree3:** Our final method, LocTree3, combines PSI-BLAST and LocTree2 in the settings where they perform best. A single parameter chooses: homology-based inference, if a profile-2-sequence PSI-BLAST hits at *E*-value ≤ 10^−3^, else: LocTree2 (‘Results’ section and Supplementary Figures S1 and S2).
4. **Public methods (CELLO 2.5, WoLF PSORT, YLoc, PSORTb 3.0):** We compared LocTree3 to four publicly available leading prediction methods: CELLO 2.5 (7), WoLF PSORT (8), YLoc (9) and PSORTb 3.0 (10). If WoLF PSORT or CELLO 2.5 predicted multiple locations, and one of those was correct, we always considered the prediction fully correct. Furthermore, these two methods distinguish cytoskeleton and cytoplasm; here, we considered both as cytosolic. Because no method other than LocTree2/3 distinguishes between membranes other than the cell membrane in eukaryotes, we merged these two classes, i.e. treated nuclear and nuclear-membrane proteins as identical. Plastid and chloroplast proteins were also merged into one class for a comparison of LocTree3 to other methods. For a comparison with CELLO 2.5 and PSORTb 3.0 we combined bacterial secreted and fimbrium proteins into one class and differentiated between Gram-positive and Gram-negative proteins according to Yu *et al.* (10).

### Reliability index

The reliability of a prediction is given through a reliability index ranging from 0 (weak prediction) to 100 (confident prediction). For LocTree2, the reliability indices are taken directly from its output. For homology-based inferences from PSI-BLAST, the reliability index was compiled as a simple function of the percentage pairwise sequence identity (PIDE) with a threshold at the saturation of PIDE ≤ 20 (Supplementary Figure S1).

### Performance evaluation

The performance for a single localization class L was expressed using accuracy (often also referred to as precision) and coverage (often also referred to as recall):

(1) \begin{equation*} {\rm Acc}({\rm L}) = 100\times \frac{{{\rm TP}}}{{{\rm TP} + {\rm PF}}} \end{equation*}

(2) \begin{equation*} {\rm Cov}({\rm L}) = 100\times \frac{{{\rm TP}}}{{{\rm TP} + {\rm FN}}} \end{equation*}

with: TP, the true positives (i.e. the number of proteins predicted and observed in localization L); FP, the false positives (i.e. the number predicted in L and observed in non-L); FN, the false negatives (i.e. the number observed in L and predicted in non-L). We measured the overall performance by the *n*-state accuracy *Qn*:

(3) \begin{equation*} {Q}n = \frac{{{\rm {\rm number}}\;{\rm {\rm proteins}}\;{\rm correctly}\;{\rm predicted}\;{\rm in}\;n\;{\rm classes}}}{{{\rm total}\;{\rm number}\;{\rm proteins}\;{\rm observed}\;{\rm in}\;n\;{\rm classes}}} \end{equation*}

Standard errors were estimated over 1000 bootstrap sets, i.e. randomly select 15% of proteins without replacement from the original data set (in our experience this non-standard procedure yields more long-lived estimates). For each bootstrap set, the performance *x_i_* (e.g. accuracy) is estimated through its difference from the overall performance $\langle x \rangle$. These 1000 estimates provided the standard deviation of *x_i_* with the typical standard error, where *n* is the number of bootstrap sets:

(4) \begin{equation*} {\rm Standard}\;{\rm deviation}\,(\sigma ) = \sqrt {\frac{{\sum\nolimits_{i = 1}^n {\left( {x_i - \langle x\rangle } \right)^2 } }}{n}} \end{equation*}

\begin{equation*} {\rm Standard}\;{\rm error} = \frac{\sigma }{{\sqrt {n - 1} }} \end{equation*}

### Runtime analysis

For sequences with pre-calculated PSI-BLAST profiles the LocTree2 runtime was measured on a Dell M605 machine with a Six-Core AMD Opteron processor (2.4 GHz, 6MB and 75 W ACP) running on Linux.

## RESULTS

### LocTree3 balanced PSI-BLAST and LocTree2

Homology-based inference for a protein of unknown localization U implies to find a protein with known localization K that is sequence similar to U (e.g. sim(U,K) > T and U ≠ K). We experimented with alternative solutions, but avoided to ‘over-optimize’. We simply chose the threshold T to be the standard PSI-BLAST *E*-value of 10^−3^ (Supplementary Figure S2, Supporting Online Material). This typically gave several hits: choosing the one with highest percentage pairwise sequence identity slightly outperformed taking the hit with best *E*-value (Supplementary Table S4).

Surprisingly, homology inference outperformed our advanced machine learning tool LocTree2 for half of our original data (995 of 1682 eukaryotic and 202 of 479 bacterial proteins, Table 1). However, when we forced PSI-BLAST to return hits for all proteins, LocTree2 consistently outperformed the PSI-BLAST protocol (Table 1).

**Table 1.** Performance for LocTree3 and its sources

Method	Eukaryota *Q*18 (Equation (3))			Bacteria *Q*6 (Equation (3))		
	Set2011_hval0 (1682)*^4^	Without PSI-BLAST hits (687) *^4^	With PSI-BLAST hits (995)*^4^	Set2011_hval0 (479)*^5^	Without PSI-BLAST hits (277)*^5^	With PSI-BLAST hits (202)*^5^
PSI-BLAST*^1^	55 ± 3	na	**93** ± **2**	40 ± 5	na	**94** ± **4**
LocTree2*^2^	65 ± 3	**61** ± **5**	67 ± 4	84 ± 4	**84** ± **5**	83 ± 6
LocTree3*^3^	**80** ± **3**			**89** ± **4**		

*Note*: ‘±’ values refer to standard errors (Equation (4)); bold face: ‘winner in each column’.

*^1^ PSI-BLAST: simple look-up of localization from proteins with known localization, excluding self-hits.

*^2^ LocTree2: *de novo* machine learning-based prediction (cross-validated).

*^3^ LocTree3: takes PSI-BLAST if available and LocTree2, otherwise.

*^4^Eukaryotic ‘Set2011_hval0’: 1682 sequence-unique eukaryotic proteins with experimental localization annotation from SWISS-PROT release 2011_04; for 995 of those, PSI-BLAST found hits at *E*-value ≤ 10^−3^ in the set of all annotations of release 2011_04, for 687 it did not.

*^5^Bacterial ‘Set2011_hval0’: SWISS-PROT release 2011_04 had localization annotations for 479 sequence-unique bacterial proteins; for 202 PSI-BLAST identified hits in the remainder of annotated proteins in 2011_04, for 227 it did not.

These first results suggested a simple protocol: use PSI-BLAST if applicable, LocTree2 if not. We dubbed the method that realized this protocol LocTree3. The combination outperformed both its sources, reaching an overall performance of *Q*18 = 80 ± 3% in classifying eukaryotic proteins in 18 classes (10 non-membrane and 8 membrane classes) and bacterial proteins in six classes at *Q*6 = 89 ± 4% (Table 1). LocTree3 predicted eukaryotic extra-cellular proteins best (Acc: 88% and Cov: 96%), followed by nuclear proteins (Acc: 81% and Cov: 86%; Supplementary Figure S3A, Supplementary Table S5). For bacteria, the prediction of plasma membrane proteins was most accurate (Acc: 96% and Cov: 95%), followed by cytosolic proteins (Acc: 91% and Cov: 90%; Supplementary Figure S3B, Supplementary Table S5).

### LocTree3 outperformed other methods

For both eukaryotes and bacteria, LocTree3 significantly outperformed its competitors on all data sets tested (Table 2 and Supplementary Table S6). Finally, we used all experimentally annotated human proteins to benchmark the methods and found LocTree3 again to provide the most accurate predictions (Supplementary Table S7). The complete human set contained 5016 proteins; LocTree3 reached *Q*10 = 89%, followed by YLoc, Cello 2.5 and Wolf Psort with 76, 75 and 71% respectively (Supplementary Table S7). LocTree3 appears best when compared on the same number of classes, and it also is the method that distinguishes in most detail with 18 classes for eukaryotes (compared to 12 for Cello 2.5 and Wolf PSORT; 11 for YLoc).

### Reliability index enables users to focus on best predictions

LocTree3 measures the confidence of each prediction through a reliability index (RI) that scales from 0 (low confidence) to 100 (high confidence). Technically, RI reflects the strength of a prediction. Our task as developers was to provide a measure that allows users to translate this strength into estimates for performance. Indeed, our RI strongly correlated with accuracy (Figure 1): when choosing the 50% most strongly predicted eukaryotic proteins, 95% of the predictions were correct (RI > 70, Figure 1: black arrow). For bacterial proteins the same level of accuracy was also reached for about half of all proteins (but at RI > 80, Figure 1: gray arrow). For users not familiar with reliability indices it is important to point out that the choice of the ‘top N’ does not require knowing the answer. Instead, any user can make this choice for any prediction and can read of Figure 1 what to expect from the choice.

### About 90 s runtime without alignment

At this point, the *PredictProtein cache* (19,20) holds >11.7 million pre-computed PSI-BLAST profiles that are quickly retrieved by LocTree3. Due to a recent acceleration of the profile kernel (16,17), the runtime of LocTree2 could be reduced by up to 100 times, such that now an average SVM kernel lookup takes about 90 s for a typical eukaryotic protein (bacteria: 4s, archaea: 2s).

Due to considerable ‘start-up’ overhead, the runtime increases sub-linearly with the number of queries. This renders the server fit for queries with entire proteomes, typically requiring few minutes for archaeal, <1 h for bacterial and <1 day for eukaryotic proteomes. If the PSI-BLAST profiles have to be created first, runtimes increase manifold, as creating a profile takes 10–500 times longer than running LocTree2. Interested users may download the LocTree3 Debian package from the web server and run it on their machines.

### Prediction workflow

Users submit one or more FASTA-formatted protein sequences. For each sequence, the server first checks for the pre-calculated results in the *PredictProtein cache*. If available, the result is returned immediately (minus queue waiting time); if not, the server retrieves a PSI-BLAST profile through the PredictProtein pipeline (19,20). The profile is used to identify hits in a database of experimentally annotated proteins. If no hits are identified, the profile triggers a *de novo* prediction by LocTree2.

For every query protein, the result contains four basic values: (i) the protein identifier as provided by the user, (ii) the reliability score of a prediction on a 0–100 scale with 100 being the most confident prediction, (iii) single predicted localization class and (iv) GO term(s) and GO identifier(s) matching the predicted class. Every result is supported by the information on whether it comes from a PSI-BLAST homology search or a LocTree2 *de novo* prediction. In case of the former, the web site provides ‘per click’ on the prediction result the experimental SWISS-PROT annotation of the best hit and its PSI-BLAST alignment to the query protein. In case of the latter, ‘the click’ on the result will forward to the visual representation of the LocTree2 decision tree and the decision path leading to the final prediction. In addition, every result is supported by a schematic representation of the biological cell highlighting the predicted localization (Figure 2).

### Predictions pre-calculated for over 1000 organisms

LocTree3 predictions for over 1000 complete eukaryotic and prokaryotic proteomes are available on the web server (http://rostlab.org/services/loctree3/proteomes/). Predictions are based on sequence sets from the European Bioinformatics Institute (EBI: http://www.ebi.ac.uk/genomes/ and http://www.ebi.ac.uk/reference_proteomes/). The high-throughput annotation and prediction of protein sub-cellular localization allows organism-wide comparisons of protein localization patterns and the reconstruction of evolutionary relations (Goldberg *et al.*, in preparation). Predictions for the newly completed proteomes will be added to the web server on a semi-annual basis.

## DISCUSSION

PSI-BLAST has certainly changed the way we do sequence analysis more than any tool (possibly excluding PubMed and Google). Furthermore, this tool has been improving continuously since its first publication in 1997 adding important value beyond that from growing databases (25). LocTree2 uses advanced SVM profile kernels (16). Although it explicitly uses local sequence similarity, LocTree2 arguably falls into the class of *de novo* methods simply because it reaches its predictions through levels of sequence similarity that are not available directly from sequence comparisons. Nevertheless, we found that a simple PSI-BLAST protocol could outperform LocTree2 for about half of the proteins in our data set (Table 1), an observation in line with the findings of Imai and Nakai (11). Unfortunately, homology-based inferences became random for the other proteins, dropping the overall average substantially below that for LocTree2 (Table 2). Thus, it would be a very bad idea to annotate an entire proteome only with homology-based inference.

**Table 2.** Performance comparison for state-of-the-art prediction methods

	Eukaryota *Q*10 (Equation (3))			Bacteria *Q*5 (Equation (3))	
Method	Set2011_hval0 (1682)*^2^	New2013_hval0 (273)*^3^	New2014 (198)*^4^	Set2011_hval0 (479)*^2^	New2013_hval0 (57)*^3^
Cello 2.5*^1^	65 ± 3	64 ± 7	81 ± 7	82 ± 4	70 ± 14
PSORTb 3.0*^1^	-	-	-	57 ± 5	51 ± 15
Wolf Psort*^1^	60 ± 3	65 ± 7	77 ± 7	-	-
YLoc*^1^	60 ± 3	63 ± 7	66 ± 8	-	-
LocTree2	65 ± 3	66 ± 7	**85** ± **6**	86 ± 4	81 ± 11
LocTree3	**81** ± **3**	**73** ± **7**	84 ± 6	**90** ± **3**	**84** ± **11**

*Note*: ‘±’ values refer to standard errors (Equation (4)); bold face: ‘winner in each column’.

*^1^Cello 2.5 (7), PSORTb 3.0 (10), Wolf Psort (8), YLoc (9) as described in ‘Materials and Methods’ section.

*^2^Set2011_hval0 (as in Table 1): 1682 sequence unique eukaryotic and 479 bacterial proteins used for development of LocTree3.

*^3^New2013_hval0: 273 eukaryotic and 75 bacterial proteins added to SWISS-PROT between releases 2011_04 and 2013_11, sequence homology reduced at HVAL < 0.

*^4^New2014: 198 eukaryotic proteins added to SWISS-PROT between releases 2013_11 and 2014_03 (not redundancy-reduced).

Our new method LocTree3 successfully navigates a path through homology-based and *de novo* prediction of localization (Tables 1-2, Supplementary Tables S5–S7, Section S2). The method is so good that it reaches 18-state overall accuracy (*Q*18, Equation (3)) >95% for half of all the proteins that are most strongly predicted, i.e. have highest reliability (Figure 1). For any new query, users can read off the results whether or not their protein is likely to fall into this top set of ‘>95%’ (RI > 70 for eukaryotes, RI > 80 for bacteria, Figure 1), and whether the prediction comes from a homology search with PSI-BLAST or a *de novo* prediction with LocTree2. For instance, LocTree3 predicts 77% of the entire proteome in human through homology-based inference (a few other highlights from Supplementary Table S8: yeast 68%, *Arabidopsis* 61%, *Caenorhabditis elegans* 47%). However, for yeast only 17% of the predictions originated from direct homology inference, the remainder came from direct experimental annotations (Supplementary Table S8). For human, the corresponding numbers were 30% experimental, 47% through homology inference (Supplementary Table S8). Unfortunately, LocTree2 cannot recover for mistakes made by the homology lookup and all our assessment is based on taking the homology lookup when available. Investigating reasons why homology-based inference was wrong did not give a clear answer (Supplementary Section S3). Due to its high overall performance, reduced prediction time and cached prediction results, LocTree3 web server optimizes well for the handling of large-scale data. Therefore, this web server and its downloadable software should provide an ideal starting point to aid the prediction of protein function through localization predictions.

## SUPPLEMENTARY DATA

Supplementary Data are available at NAR Online.

Supplementary Data
