# Supplementary material for: LocTree3 prediction of localization
Source: Nucleic Acids Res. 2014 May 21;42(Web Server issue):W350–5. doi: 10.1093/nar/gku396 (PMC4086075; doi:10.1093/nar/gku396)
Supplement: Supplementary Data [file supp_42_W1_W350__index.html]

Supplementary Data 

# LocTree3 prediction of localization

## Supplementary Data

**Files in this Data Supplement:**

- SUPPLEMENTARY DATA
