# Supplementary material for: LocTree3 prediction of localization
Source: Nucleic Acids Res. 2014 May 21;42(Web Server issue):W350–5. doi: 10.1093/nar/gku396 (PMC4086075; doi:10.1093/nar/gku396)
Supplement: Supplementary Data [file supp_gku396_nar-00423-web-b-2014-File004.pdf]

Supporting online material for: LocTree3 prediction of localization

Tatyana Goldberg, Maximilian Hecht, Tobias Hamp, Timothy Karl, Guy Yachdav, Henrik Nielsen, Burkhard Rost & et al.

Table of Contents for Supporting Online Material

Table S1: Data set for development and evaluation	2
Table S2: Homology-based inference from sequence-unique sets	3
Table S3: Data sets for independent/additional testing	4
Section S1: LocTree3 assessment on multi-localized proteins	5
Figure S1: PSI-BLAST sequence identities to LocTree3 reliability scores	6
Figure S2: E-value thresholds for the homology-based inference	7
Table S4: Strategies for annotation transfer by homology	8
Figure S3: Class-wise performance comparison of LocTree3 to its sources	9
Table S5: LocTree3 assessment on sequence-unique sets	10
Table S6: Performance comparison on LocTree3's development data	11
Table S7: Performance comparison on a human protein set	12
Table S8: Proteome-wide localization predictions using PSI-BLAST	12
Section S2: LocTree3 is much more reliable than blind homology-inference	13
Section S3: Possible sources for PSI-BLAST mis-predictions	14

Material

Table S1: Data sets for development and evaluation.

Localization	*Eukaryota*	*Bacteria*	*Archaea*
Chloroplast	133	-	-
Chloroplast membrane	11	-	-
Cytosol	220	179	41
Endoplasmic reticulum	10	-	-
Endoplasmic reticulum membrane	65	-	-
Extra-cellular space	596	82	5
Fimbrium	-	16	-
Golgi apparatus	3	-	-
Golgi apparatus membrane	17	-	-
Mitochondria	140	-	-
Mitochondria membrane	87	-	-
Nucleus	320	-	-
Nucleus membrane	5	-	-
Outer membrane	-	6	-
Plasma membrane	40	144	13
Periplasm	-	52	-
Peroxisome	6	-	-
Peroxisome membrane	2	-	-
Plastid	14	-	-
Vacuole	3	-	-
Vacuole membrane	10	-	-
SUM	1682	479	59

Data: number of proteins per localization class with experimentally determined annotations of a single subcellular localization taken from SWISS-PROT release 2011_04 (1) in at HVAL≤0 (2, 3) sequence-unique sets of eukaryotic, bacterial and archaeal proteins. The data sets were used for development of LocTree3 and its predecessor LocTree2 (4).

Table S2: Homology-based inference from sequence-unique sets

PSI-BLAST E-value threshold[*1]	Q18 - Eukaryota (1682 proteins)	Q6 - Bacteria (479 proteins)
10^{-7}	1±1	0
10^{-5}	2±1	0.4±1
10^{-3}	5±1	2±2
10^{-1}	17±2	9±3
1	27±3	25±5
10	32±3	39±6
100	24±2	33±5
100000	22±2	28±5
Random[*2]	22±2	28±5

Data: 1682 eukaryotic and 479 bacterial sequence-unique proteins with an experimental annotation of a single sub-cellular localization extracted from SWISS-PROT release 2011_04, aligned against themselves.

1* PSI-BLAST E-value threshold: defines the E-value (5, 6) threshold for a PSI-BLAST (7) hit, which is different to the query protein, to be considered for performance evaluation

*2 Random: defines the performance of a random prediction in one of eighteen classes in Eukaryota and six classes in Bacteria, with respect to the data distribution among these classes

Note: Q is the overall prediction accuracy (Eqn. 3, Methods); "±" values refer to standard errors (Eqn. 4, Methods)

Table S3: Data sets for independent/additional testing.

Localization	*New2013_hval0*[*1]		*New2014*[*2]	*Human*[*3]
	Eukaryota	*Bacteria*	*Eukaryota*	*Eukaryota*
Chloroplast	10	-	8	-
Chloroplast membrane	14	-	-	-
Cytosol	43	19	25	965
Endoplasmic reticulum (ER)	1	-	1	41
ER membrane	7	-	-	175
Extra-cellular space	112	20	121	744
Fimbrium	-	1	-	-
Golgi apparatus	2	-	2	15
Golgi apparatus membrane	4	-	-	83
Mitochondrion	13	-	1	290
Mitochondrion membrane	7	-	-	112
Nucleus	43	-	34	1524
Nucleus membrane	-	-	-	7
Outer membrane	-	4	-	-
Periplasm	-	5	-	-
Plasma membrane	9	8	6	1020
Peroxisome	1	-	-	25
Peroxisome membrane	1	-	-	13
Plastid	-	-	-	-
Vacuole	1	-	-	-
Vacuole membrane	5	-	-	2
SUM	273	57	198	5016

Data: number of sequences per localization class in the sets of SWISS-PROT proteins used for the independent/additional testing of LocTree3.

[*1] "New2013_hval0" set: at HVAL≤0 redundancy reduced sets of 273 eukaryotic and 57 bacterial proteins, thus containing no protein pair with >20% pairwise sequence identity over 250 residues aligned. Redundancy reduced set of archaeal proteins was too small (18 proteins) to provide meaningful performance estimates and was thus excluded from the analysis.

[*2] "New2014" set: all eukaryotic proteins added to SWISS-PROT between releases 2013_11 and 2014_03, not redundancy reduced. Because the number of corresponding bacterial proteins was too small (10 proteins), they were excluded from the analysis.

[*3] "Human" set: all proteins with an experimental annotation of exactly one localization class in the SWISS-PROT release 2014_03, not redundancy reduced.

Section S1: LocTree3 assessment on multi-localized proteins

LocTree2 and LocTree3 were developed on proteins from the Swiss-Prot release 2011_04. The number of multi-localized proteins in this release was 48 for bacteria (all annotated with two localization classes) and 4556 for eukaryota (4376 with two localization classes, the others with ≥3). Due to the small number, we dropped bacteria. Reducing redundancy at HVAL≤0 on these 4556 left us with 72 sequence-unique proteins. We applied LocTree3 to these and considered the prediction correct if one of the experimentally observed classes had been predicted. Result: Q18=65±12%; while similar to the performance of LocTree2 on the 1682 cross-validate proteins, it compared less favourable to 80±3% for LocTree3. Why did performance drop on those proteins? Clearly, the random expectation was the opposite, i.e. since we allow one mistake we have a higher random performance: picking one right from 18 is tougher than picking 2 and choosing the best-of-two. In short, our suspicion is that today's double annotations as a whole set are not good enough.

We looked at LocTree3 predictions for five misclassified proteins (i.e. proteins for which none of the experimentally annotated localization classes could be picked by LocTree3) with the highest reliability scores (RIs). One of the five proteins (YG4O_YEAST, RI=38) was an uncharacterized protein while for the remaining four we were able to find the experimental evidence for the predicted localization classes in other sources rather than Swiss-Prot: (1) ZYM1_SCHPO is a metallothionein, which is annotated to be localized to the nucleus and the cytoplasm in SWISS-PROT. LocTree3 predicts this protein to be secreted with the RI=98, we found an experimental evidence for metallothioneins to be secreted in Moltedo *et al.* (8); (2) GPX41_MOUSE is annotated to localize to the mitochondrion and the cytoplasm, while LocTree3 predicts nucleus with the RI=93, which is confirmed by Yant *et al.* (9); (3) NPC2_ASPOR is annotated to be cytoplasmic and a Golgi apparatus protein, LocTree3 however predicts it to be vacuolar with the RI=43, which is true for the protein's ortholog NPC2_YEAST; (4) PEN2_CAEEL is annotated to be localized to the ER membrane and Golgi membrane, LocTree3 predicts mitochondria membrane with RI=36 which is true according to the work of Hansson *et al.* (10)*.* Interestingly, for the protein with the lowest prediction reliability index (CSN4_BRAOL, RI=6) and the predicted localization class chloroplast we could find an evidence in Xiangjun *et al.* (11) stating that the protein acts as a suppressor of chloroplast development. SWISS-PROT annotates the protein to be nuclear and cytoplasmic.

From these findings we conclude that the number of sequence-unique multi-localized proteins as we have them today in SWISS-PROT is rather small and the annotations of multiple localization may be fuzzy and incomplete. Therefore, assessing prediction methods on these proteins may lead to underestimated results and incorrect implications.

Figure S1:

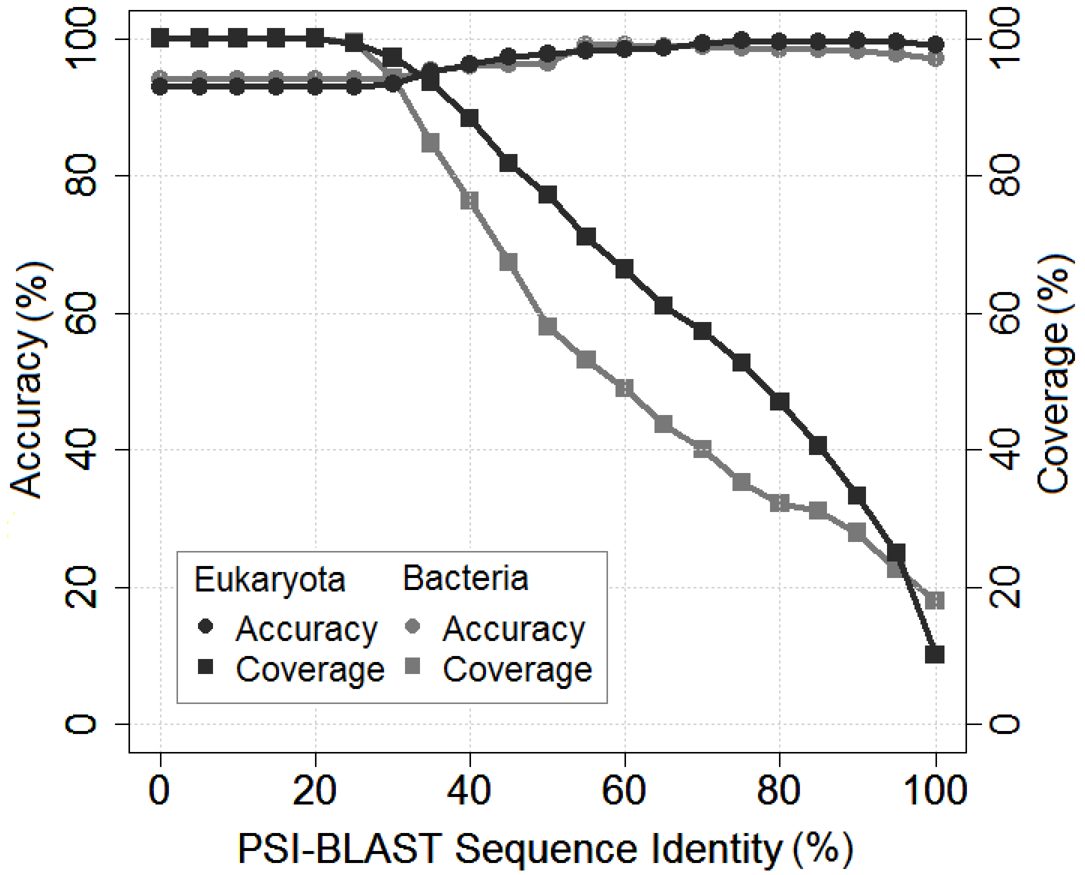

Fig. S1: PSI-BLAST sequence identities to LocTree3 reliability scores. Localization annotation from sequence homologs is more accurate at higher PSI-BLAST pairwise sequence identity (PIDE) values. Here we show the percentage Accuracy/Coverage (Methods) at the given sequence identity thresholds for 995 eukaryotic and 202 bacterial proteins that had a PSI-BLAST hit with E-value≤10^{-3} (6, 7). Since method's performance did not change for PIDE<20, we formed LocTree3's reliability index by normalizing the sequence identity values according to (PIDE-20)*10/8.

Note, the slight decrease of the Accuracy curves at PIDE approaching 100% results from the changed annotations in SWISS-PROT between releases 2011_04 and 2013_11. Though these proteins are predicted to be localized correctly in 2013_11, they are considered as false predictions in the current evaluation (Eukaryota: AIM37_YEAST, ECP_MACFA; Bacteria: ESPR_MYCTU).

Figure S2:

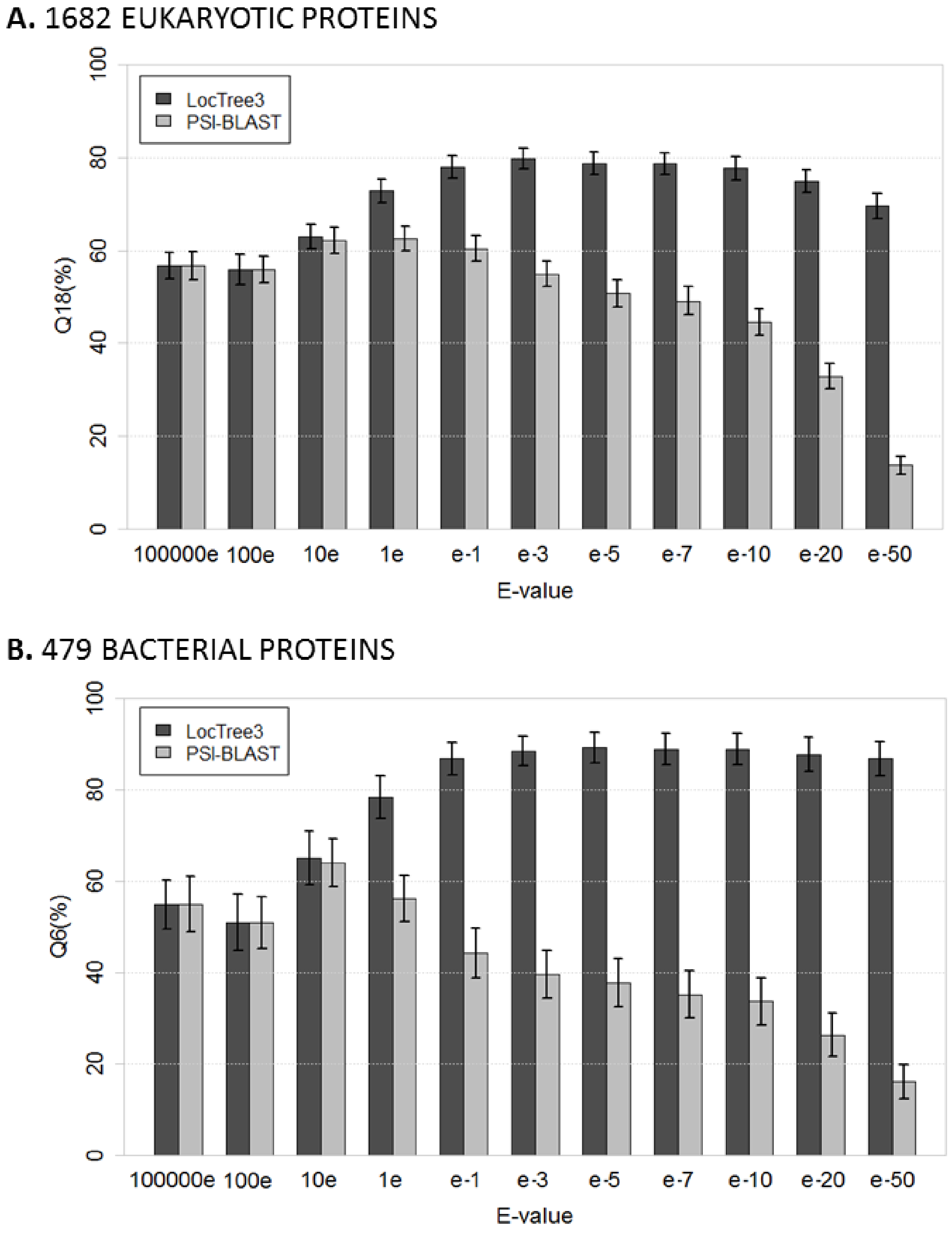

Fig. S2: E-value thresholds for the homology-based inference from all experimentally annotated proteins in SWISS-PROT release 2011_04

The accuracy of localization annotation transfer from sequence homologs (entire SWISS-PROT release 2011_04: 34583 eukaryotic and 4765 bacterial proteins) varies at different PSI-BLAST E-values. Shown is the overall accuracy of LocTree3 (dark grey) and PSI-BLAST (light grey) in predicting 18 localization classes (Q18, Methods) for eukaryotes (Panel A) and 6 classes for bacteria (Panel B) at the given E-value cut-off. PSI-BLAST E-value thresholds reached their peak at high E-value≤10. However, in order to determine the threshold at which value to use LocTree2 and at which PSI-BLAST, we also need to consider the performance of the final merger LocTree3 at the same threshold. The optimal threshold for LocTree3 seemed to be much more conservative, namely at E-value≤10^{-3}.

Table S4: Strategies for annotation transfer by homology.

Method / *Performance*		*Minimum E-val*	*Maximum HVAL*	*Maximum PIDE*	*Majority vote*
Euka-ryota	*Q(18), 1682 proteins*	54 ± 3	53 ± 3	**55 ± 3**	53 ± 3
Bac-teria	*Q(6), 479 proteins*	**40 ± 6**	38 ± 5	**40 ± 5**	39 ± 5

Data: sequence-unique sets of 1682 eukaryotic and 479 bacterial proteins extracted from SWISS-PROT release 2011_04. For each protein a PSI-BLAST profile was built using a combination of UniProt (1) and PDB (12) databases redundancy reduced at 80% sequence identity. The profiles were then aligned at the standard E-value of 10^{-3} (6, 7) against 34583 experimentally annotated eukaryotic and 4765 bacterial proteins available in SWISS-PROT in 2011_04. Given a list of homologs for a query protein we investigated which of the following strategies contributed most to the overall performances Q18 (i.e. correct classification of a protein in one of 18 classes) for Eukaryota and Q6 (i.e. correct classification of a protein in one of 6 classes; Methods) for Bacteria:

Minimum E-val: take the annotation of the hit with the minimum expectation value

Maximum HSSP-val: take the annotation of the hit with the maximum HVAL (9, 10)

Maximum PIDE: take the annotation of the hit with the maximum pairwise sequence identity

Majority vote: take the localization class of most hits

When more than one hit fit the same (e.g. maximum PIDE), we picked the first.

Figure S3:

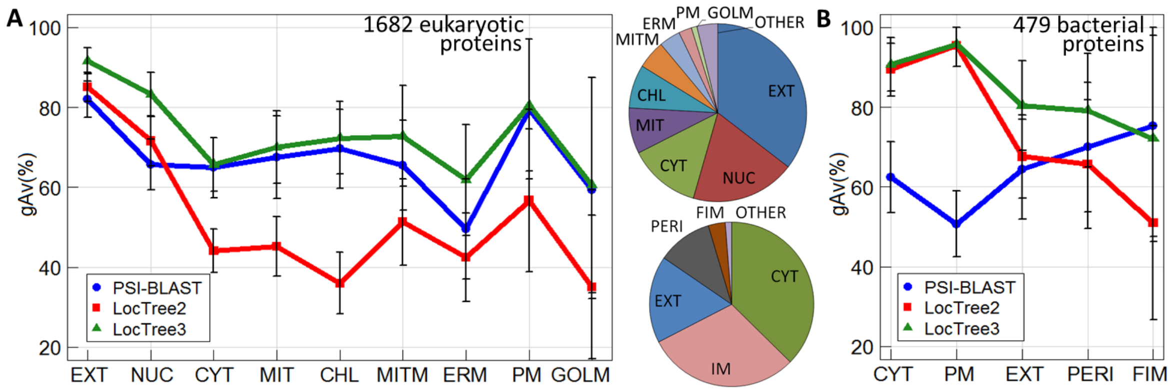

Fig. S3: Class-wise performance comparison of LocTree3 to its sources

PSI-BLAST marks a simple 'lookup' in the database of experimentally annotated proteins from the SWISS-PROT release 2011_04 (i.e. 34583 eukaryotic and 4765 bacterial proteins), self-hits are excluded; LocTree2 is a *de novo* machine learning-based predictor, results shown here are valid for cross-validation on 1682 eukaryotic and 479 bacterial proteins. LocTree3 combines the results of previous two methods by taking PSI-BLAST hits with E-value≤10^{-3} and maximum PIDE, if available, and LocTree2 predictions otherwise. We tested on a non-redundant data set of (A) 1682 eukaryotic and (B) 479 bacterial proteins extracted from SWISS-PROT release 2011_04. The localization classes (compartments) on the x-axes mark the averages over all proteins in that class. Note that the x-axes are sorted by the prevalence of that class in the experimental annotations (as given by the inlet pie-charts). In this graph, we force PSI-BLAST to always return a prediction. The y-axes show the geometric average (gAv, Methods) between accuracy and coverage. The pie charts in the centre show the fraction of proteins belonging to each class. LocTree2 predicted classes with most experimental annotations best (A: EXT+NUC, B: CYT+IM+EXT). We could not confirm the same trend for the simple PSI-BLAST protocol. Overall, our new method, LocTree3, published in the web server still maintains a small correlation between performance and experimental annotations with respect to the compartments.

Abbreviations: gAv, geometric average; CHL, chloroplast; CYT, cytosol; ERM, endoplasmic reticulum membrane; EXT, extra-cellular; FIM, fimbrium; GOLM, Golgi apparatus membrane; MIT, mitochondria; MITM, mitochondria membrane; NUC, nucleus; PERI, periplasmic space; PM, plasma membrane.

Table S5: LocTree3 assessment on sequence-unique sets of 479 bacterial and 1682 eukaryotic proteins

Localization	*Nprot*	*Acc*	*Cov*	*gAv*
Extra-cellular	596	88 ± 3	96 ± 2	92 ± 4
Nucleus	320	81 ± 5	86 ± 5	83 ± 6
Cytosol	220	68 ± 7	64 ± 8	66 ± 7
Mitochondria	140	74 ± 10	66 ± 10	70 ± 8
Chloroplast	133	72 ± 9	73 ± 10	72 ± 9
Mitochondria membrane	87	77 ± 11	69 ± 11	73 ± 11
ER membrane	65	67 ± 16	57 ± 14	62 ± 13
Plasma membrane	40	84 ± 15	78 ± 16	81 ± 16
Golgi membrane	17	69 ± 31	53 ± 29	61 ± 27
Plastid	14	50 ± 50	29 ± 31	38 ± 23
Chloroplast membrane	11	80 ± 29*	73 ± 29*	76 ± 32*
ER	10	71 ± 47*	50 ± 35	60 ± 33
Vacuole membrane	10	100*	40 ± 31	63 ± 32
Q(18) – Eukaryota	1682	80 ± 3		
Cytosol	179	91 ± 5	90 ± 5	91 ± 7
Plasma membrane	144	96 ± 4	95 ± 4	96 ± 5
Extra-cellular	82	75 ± 11	87 ± 9	80 ± 11
Periplasm	52	82 ± 14	77 ± 14	79 ± 15
Fimbrium	16	83 ± 25*	63 ± 35	72 ± 26
Q(6) – Bacteria	479	89 ± 4		

Data sets and the LocTree3 performance estimation as in Figure S3. Abbreviations used: *Nprot*, the number of proteins with known localization; *Acc*, accuracy; *Cov*, coverage; *gAv*, geometric coverage of *Acc* and *Cov*; *Q(n)*, overall prediction accuracy. Standard errors were estimated by bootstrapping (Methods).
Note 1: *Q(n)* is a six-state value for bacteria, i.e. the overall accuracy for classification in one of six localization classes, and an eighteen-state value for Eukaryota (Methods). Note 2: Only performances for localization classes containing more than ten proteins are reported.
* = unrealistic upper or lower bound given by the standard error due to the small data set size.

Table S6: Performance comparison on LocTree3's development data

Method		Eukaryota "Complete" set (1682)[*7]	Eukaryota ¬PSI-BLAST hits (687)[*7]	Eukaryota PSI-BLAST hits (995)[*7]		Bacteria "Complete" set (479)[*8]	Bacteria ¬PSI-BLAST hits (277)[*8]	Bacteria PSI-BLAST hits (202)[*8]
Cello 2.5[*1]	Q10	65±3	60±5	70±4	Q5	82±4	81±5	83±6
PSORTb 3.0[*2]		-	-	-		57±5	47±7	71±7
Wolf Psort[*3]		60±3	57±5	63±3		-	-	-
YLoc[*4]		60±3	55±5	64±4		-	-	-
LocTree2[*5]		65±3	62±4	68±4		86±4	86±5	85±6
LocTree3*[6]		**81±3**	**62±4**	**94±2**		**90±3**	**86±5**	**94±4**

[*1] Cello 2.5: employs a system of Support Vector machines to classify eukaryotic proteins in 12 and bacterial in 5 classes using sequence-derived features (13)

[*2] PSORTb 3.0: predicts four classes for Gram-positive and five classes for Gram-negative bacteria through a combination of several classifiers into a Bayesian network (14)

[*3] Wolf Psort: k-nearest neighbour classifier that predicts 12 localization classes for eukaryotes from sequence-derived features (15)

[*4] YLoc: uses sequence-derived features together with GO terms to classify eukaryotic proteins in 11 localization classes through Naïve Bayes (16)

[*5] LocTree2: *de novo* machine learning-based method, results valid for cross-validation

[*6] LocTree3: combines *de-novo* (LocTree2) and homology-based (PSI-BLAST) searches; it uses PSI-BLAST predictions (lookup at E-value$\leq 10^{-3}$ in a database of experimentally annotated proteins) if available and LocTree2 (results from the cross-validation setting), otherwise

[*7] data set Eukaryota: 1682 sequence-unique eukaryotic proteins in SWISS-PROT release 2011_04; for 995 of those we found PSI-BLAST hits, for 687 we did not

[*8] data set Bacteria: 479 sequence-unique bacterial proteins in SWISS-PROT release 2011_04; for 202 of those we found PSI-BLAST hits, for 227 we did not

Note: Q is the overall prediction accuracy (Eqn. 3, Methods); "±" values refer to standard errors (Eqn. 4, Methods); bold face: "winner in each column"

Table S7: Performance comparison on human protein data

Method	*Q10 (Eqn. 3, Methods) "Human proteins" set (5016)*[*6]
Cello 2.5[*1]	75±1
Wolf Psort[*2]	71±1
YLoc[*3]	76±1
LocTree2[*4]	76±1
LocTree3[*5]	**89±1**

[*1-5] Methods as in Table S6

[*5] data set "Human proteins": 5016 human proteins with an experimental annotation of exactly one localization class in SWISS-PROT release 2014_03. A vast majority of these proteins constitutes the training sets of the methods tested.

Note: "±" values refer to standard errors (Eqn. 4, Methods); bold face: "winner in each column"

Table S8: Proteome-wide localization predictions using PSI-BLAST

Organism name	#proteins predicted[*1]	#PSI-BLAST predictions[*2] (% in relation to #proteins predicted)	#Self-hits[*3] (% in relation to #PSI-BLAST predictions)
H. sapiens	20249	15671 (77%)	4638 (30%)
S. cerevisiae	6434	4372 (68%)	2209 (51%)
A. thaliana	27270	16527 (61%)	1843 (11%)
C. elegans	20791	9780 (47%)	346 (4%)
B. weihenstephanensis	5650	1862 (33%)	1 (<1%)
A. pernix	1700	133 (8%)	2 (1%)

[*1] number proteins predicted with LocTree3 in the proteomes of six completely sequenced organisms downloaded from http://www.ebi.ac.uk/genomes/

[*2] number of proteins predicted by PSI-BLAST. The numbers in parenthesis are fractions in relation to the total number proteins predicted in an organism

[*3] number of PSI-BLAST self-hits, i.e. hits that were identical to query proteins. The numbers in parenthesis are fractions in relation to the total number proteins predicted by PSI-BLAST

Section S2: LocTree3 is much more reliable than blind homology-inference. Two recent advances in molecular biology make it impossible to blindly trust annotations. The first are high-throughput experiments that typically change the value of an annotation from, e.g. “protein Q is native in the Golgi” to “protein Q has been detected to have entered the secretory pathway with a probability of 0.7”. Clearly, using the second statement to annotate Q as extra-cellular would be very wrong. But what if we added “secretory pathway” as a new “class”, should we then annotate it as in that class, or should we maintain the probability? If we maintained the probability: should this be counted as “localization annotated”? What about a protein Q2 that is sequence similar to Q: should we annotate its localization also to be “secretory pathway with 70% chance”? One simple experimental data point generates so many questions that cannot be answered without generating new problems! Thousands of such data points are being created by modern molecular biology every month.

The second problem is contained in the first, but much more prevalent in today’s databases that are still heavily based upon detailed biochemical experiments. Assume that we have a reliable annotation for Q as Golgi: how to treat proteins that are related to Q? For instance, those related in terms of sequence similarity. This brings up the argument of Imai & Nakai (17), namely that PSI-BLAST predicts localization more accurately than *de novo* methods. Here we showed that this is true to some extent (Table 1: for some proteins PSI-BLAST is better than LocTree2), but that if predictions are forced, the opposite becomes true (Table 1: averaged over all proteins PSI-BLAST is much less accurate than LocTree2). Clearly, the tool we make available now, LocTree3, settles the discussion. Even if we were right that LocTree3 is the best method currently available to predict protein localization, should we apply it to annotate localization in databases that are exclusively based on experiments such as SWISS-PROT (1)? We suggest a negative answer: leave experimental annotations as clean as possible. Should we then remove almost 90% of (stand Feb. 2014) all annotations about localization in SWISS-PROT (i.e. those based on non-experimental findings)? What about a database that pulls in automated annotations such as UniProt and/or GO (18)? Naïve users querying UniProt might get the impression that over 5m (million) proteins have annotations for localization when the best we can do to develop prediction methods is dig out a list of may be 25k (thousand), i.e. 200 times fewer than suggested by that naïve sieve through UniProt. Clearly, we argue that it would be better to remove the 5m-25k inferred annotations and replace those by LocTree3 predictions marked as predictions and by possibly augmenting this with predictions for all other 45m proteins in today’s UniProt (total 52m in Feb. 2014).

Section S3: Possible sources for PSI-BLAST mis-predictions

The idea behind LocTree3 is to use PSI-BLAST if it finds hits and LocTree2, otherwise. Thus if a prediction of the sub-cellular localization is incorrect and is derived from PSI-BLAST, it cannot be ‘corrected’ by LocTree2 anymore.

Nevertheless, we looked into the cases for which PSI-BLAST annotated proteins incorrectly. In our development eukaryotic data of 1682 eukaryotic proteins, 995 proteins were classified by PSI-BLAST and for remaining 687 proteins it failed to identify a homolog in the data set of all experimentally annotates proteins. Of 995 predicted proteins 69 were misclassified. The most commonly mis-classified pairs of classes (one observed, the other looked up from homolog) were: mitochondria and chloroplast (9 times), plastid and chloroplast (8 times), cytoplasm and nucleus (8 times), cytoplasm and secreted (6 times), cytoplasm and mitochondria (5 times).

These pairs either resembled compartments that are either close in space (e.g. cytoplasm and nucleus), closely related (chloroplasts present one of the three types of plastid) or are very similar in their structure (chloroplast and mitochondria). Therefore, the PSI-BLAST mis-classifications may originate from incorrect experimental annotations, as well as from similarity in translocation signals. About 33% of the mistakes originated from “honest orthologs” (e.g. RK32_EUGGR annotated chloroplast but predicted plastid as its ortholog RK32_ASTLO). The mis-classification with the highest score (PIDE=88%) was made for ECP_MACFA, a protein for which the SWISS-PROT has changed since LocTree3 development from cytosol to be secreted, the latter correctly identified by PSI-BLAST. In other word, this mistake was based on an incorrect earlier annotation.

References for Supporting Online Material

1. A. Bairoch and R. Apweiler, *Nucleic acids research*, **28**, 45-48. (2000)
2. B. Rost, *Protein engineering*, 85-94 (1999)
3. C. Sander, R. Schneider, *Proteins*, 56-68 (1991)
4. T. Goldberg, *et al.*, *Bioinformatics.* **28**, i458-i465 (2012)
5. D. Przybylski and B. Rost, *Proteins*, **46:**197-205 (2002)
6. S.F. Altschul and W. Gish, *Methods in enzymology*, **266:**460-480 (1996)
7. S.F. Altschul, *et al., Nucleic acids research*, **25:**3389-3402 (1997)
8. O. Moltedo, *et al.*, *The Journal of biological chemistry*, **275**, 31819-31825 (2000)
9. L.J. Yant, *et al.*, Free radical biology & medicine, **34**, 496-502 (2003)
10. C.A. Hansson, *et al.*, The Journal of biological chemistry, **279**, 51654-51660 (2004)
11. X. Zhou, *et al.*, BMC plant biology, **11**, 169 (2011)
12. H.M. Berman, *et al., Nucleic acids research*, **28**(1):235-242 (2000)
13. C.S. Yu, *et al., Proteins*, **64**, 643-651 (2006)
14. N.Y. Yu, *et al.*, Bioinformatics, 26, 1608-1615 (2010)
15. P. Horton, *et al.*, Nucleic acids research 2007, 35, W585-W587 (2007)
16. S. Briesemeister, *et al., Bioinformatics* 2010, **26**, 1232-1238 (2010)
17. K. Imai and K. Nakai, *Proteomics* 2010, **10**, 3970-3983 (2010)
18. E.C. Dimmer, *et al., Nucleic acids research* 2012, **40**, D565-570 (2012)
